# Operationalizing One Digital Health and FAIR Data Principles for Disease Surveillance in a Low-Medium Income Country in Africa: Qualitative Study in the Democratic Republic of the Congo

**DOI:** 10.2196/93321

**Published:** 2026-08-05

**Authors:** Harvey Basivikidi, Lefilso'o Lambuku, Delphin Kayembe, Arriel Benis, Didier Bompangue

**Affiliations:** 1 One Health Institute for Africa University of Kinshasa Kinshasa, Kinshasa the Democratic Republic of the Congo; 2 Department of Anthropology University of Kinshasa Kinshasa, Kinshasa the Democratic Republic of the Congo; 3 Department of Biomedical Engineering Duke University Durham, NC United States; 4 Department of Biomedical Informatics Jacobs School of Medicine and Biomedical Sciences University at Buffalo Buffalo, NY United States; 5 Chrono-Environnement, UMR CNRS 6249 Université de Franche-Comté Besançon, Bourgogne France

**Keywords:** One Digital Health, One Health, FAIR principles, digital health, data interoperability, data governance, zoonotic disease surveillance, qualitative research, Democratic Republic of Congo, lower-middle-income country

## Abstract

**Background:**

Recent crises involving zoonotic diseases (Ebola, COVID-19, and Mpox) have highlighted the limitations of fragmented public health systems for the prevention and response to health emergencies at the international and continental level, and particularly in Africa, more specifically in the Democratic Republic of the Congo (DRC). In this context, the One Health (OH) approach and its extension, One Digital Health (ODH), articulated with the findable, accessible, interoperable, reusable (FAIR) principles, offer a framework for rethinking the digital transition in health in the DRC. This study analyzes the reality of this transition in Kinshasa, DRC, and questions the feasibility of ODH in this context.

**Objective:**

This study aimed to (1) explore how stakeholders across human, animal, and environmental health sectors perceive and experience digital tool integration and data interoperability in Kinshasa; (2) identify structural, institutional, and technical constraints affecting cross-sectoral data sharing; and (3) analyze the sociotechnical conditions required for the operationalization of ODH in a fragmented digital health (DH) context.

**Methods:**

A qualitative study was conducted in Kinshasa, DRC, between November 10, 2025, and November 25, 2025, combining semistructured interviews with key actors (health professionals, administrative officials, digital experts, and engaged citizens) and a document review of strategic and regulatory texts related to DH in the DRC. The data were analyzed using a thematic approach to identify the representations, uses, and constraints related to DH and the operationalization of ODH.

**Results:**

Overall, 22 stakeholders participated (n=9, 40.9% human health; n=8, 36.4% animal health; n=4, 18.2% environmental health; n=1, 4.50% digital sector), predominantly male (n=15, 68.2%) and mainly in operational roles (n=12, 54.5%). Three interrelated topics emerged. First, a dual-track digital ecosystem characterized by the coexistence of formal platforms (eg, District Health Information Software 2 and electronic records) and informal tools (eg, WhatsApp [Meta]), with persistent paper-digital double-entry generating inefficiencies. Second, structural and governance bottlenecks, including electricity instability, limited connectivity, software incompatibility, external data-hosting concerns that affect sovereignty, and institutional silos that privilege human health over animal and environmental sectors. Third, prerequisites for operationalizing ODH emphasize foundational infrastructure (energy and internet), sustainable capacity building beyond one-off training, interoperable “bridges” between fragmented systems, and high-level political leadership. These elements were synthesized into an ODH-FAIR DRC conceptual model structured around three enabling pillars linking sectors for integrated zoonotic surveillance.

**Conclusions:**

Operationalizing ODH in DRC requires addressing foundational enablers beyond tools: synchronized energy-digital policies, decompartmentalized governance, and context-adapted capacity building. These insights inform low- and middle-income countries’ DH strategies, urging donors and ministries to prioritize interoperability over isolated pilots to achieve sustainable zoonotic surveillance.

## Introduction

### Background

In recent decades, we have seen a notable increase in emerging and reemerging diseases, such as the West and Central Africa Ebola outbreak (2014-2016) [1], the COVID-19 pandemic (2019-2023) [2], and Mpox epidemics (2022-2026) [3]. Emerging and reemerging infectious diseases, largely of zoonotic origin, are now a growing threat to health systems, particularly in low- and middle-income countries (LMICs) [1-3]. These dynamics are reinforced by environmental, climatic, and urban transformations, which complicate interactions between ecosystems and human and animal populations [4,5]. In this context, the One Health (OH) approach has emerged as a strategic framework to promote integrated cross-sectoral and multisectoral action to optimize human, animal, and environmental health [6,7].

At the same time, digital health (DH) strengthens health systems, improves surveillance, supports decision-making, and contributes to universal health coverage goals [8,9]. However, in sub-Saharan Africa, DH remains fragmented, with isolated and unsustainable initiatives [10-12]. Several studies have shown that the introduction of digital tools, when not accompanied by solid governance and a systemic vision, can lead to “e-chaos” rather than to a real strengthening of health systems [11]. Nevertheless, despite these developments, the interactions between fragmented DH systems and the multidimensional and multisectoral ambitions of OH remain insufficiently explored.

In this context, One Digital Health (ODH) extends OH by linking digital technologies, data governance, and cross-sector collaboration to support integrated health action [13-15]. ODH is based on three interconnected digital spheres (health at the individual level, population-based, and environmental issues), as well as on five cross-cutting areas focused on uses: (1) education, (2) civic engagement, (3) environmental monitoring, (4) health of humans and animals as a whole, and (5) standardization, interoperability, and digitalization. On the other hand, ODH advocates for and encourages the development and implementation of data governance based on the findable, accessible, interoperable, reusable (FAIR) principles [16,17]. This approach aims to go beyond simple sectoral digitalization to build digital ecosystems that support interdisciplinary collaboration and the production of transversal knowledge.

In the Democratic Republic of the Congo (DRC), issues related to DH and the OH approach are explicitly recognized in several national strategic frameworks, including the National Health Information Systems Development Plan (PNDIS II) [18], DHIS2 (District Health Information Software 2) initiatives [19], as well as the overall orientations of the National Strategic Development Plan [20]. However, existing studies highlight that health information systems remain poorly interoperable, insufficiently integrated across sectors, and highly dependent on external technical solutions [21,22].

Recent years have also been marked by growing digitalization efforts across multiple sectors in the DRC. Particularly, in the human health sector, the Integrated Disease Surveillance and Response 3rd edition (IDSR; in French, “Surveillance Intégrée de la Maladie et Riposte”) [23] explicitly promotes the use of digital tools for surveillance and reporting. In the environmental sector, initiatives led by the Ministry of Environment and the Congolese Institute for Nature Conservation (ICCN; in French, “Institut Congolais pour la Conservation de la Nature”), often implemented with international technical and financial support, aim to strengthen environmental monitoring and data management. In animal health, the Integrated Animal Disease Surveillance and Response (IADSR; in French, “Surveillance Intégrée des Maladies Animales et Riposte”) framework similarly supports digital approaches for disease surveillance [24]. However, important challenges persist in translating these initiatives into routine operational practice to mitigate the remaining visible fragmentation within sectors (intrasectoral interoperability) and across sectors (intersectoral interoperability), limiting integrated digital governance.

In addition, the local context of a low-income country with a gross domestic product (GDP) per capita of US $649.40 [25] is characterized by significant structural constraints, including an unstable electricity supply, poor internet connectivity, and uneven penetration of digital technologies, especially outside major urban centers [26]. Human and organizational challenges, such as low digital literacy, reinforce these infrastructural limitations [27], resistance to technological change [28], and concerns about data security and sovereignty [15,17].

Although the DRC has significant experience implementing the OH approach, particularly in managing zoonoses and health emergencies [29], few studies have empirically explored the relationship between OH and DH from an integrated perspective. In particular, there is a lack of qualitative data on how actors perceive digital tools, their daily use, the institutional conditions for their adoption, and the obstacles to operationalizing an ODH approach in the DRC context.

While DH in the human health sector has been relatively well documented in DRC, much less is known about digital practices, constraints, and expectations that align or diverge across the animal and environmental health sectors. In this regard, ODH should not be understood as a simple extension of existing human DH systems, but rather as a multisectoral framework that highlights systemic fragmentation and coordination challenges across sectors. However, empirical evidence on how such integration is perceived and operationalized in real-world low-resource settings remains limited.

### Aims, Objectives, and Goals

The primary aim of this study was to explore how stakeholders across human, animal, and environmental sectors perceive and experience the integration of digital tools in the DRC context.

Specifically, the study objectives were to (1) examine how digital practices and constraints differ or align across sectors; (2) identify structural, institutional, and technical barriers to cross-sectoral interoperability; and (3) analyze the sociotechnical conditions required for the operationalization of ODH in a fragmented DH environment.

By using a qualitative methodology comprising semistructured interviews and a targeted literature review anchored in the ODH conceptual framework, our main goal was to develop a contextualized understanding of the challenges inherent in integrated DH [13,15].

This study does not presuppose ODH’s success; rather, it seeks to identify the enabling conditions and barriers that will determine whether such a framework can be meaningfully operationalized in a low-resource, multisectoral context. Ultimately, the overarching goal of this work was to provide a robust empirical evidence base to inform public policies and enhance data governance, thereby promoting the development of more coherent, sustainable, and equitable DH ecosystems in LMICs, with a specific focus on the needs of sub-Saharan Africa. The main research question was: “How do key stakeholders across human, animal, environmental, and digital sectors in Kinshasa perceive and use DH tools, and what conditions do they identify as necessary to operationalize ODH and FAIR data principles?”

## Methods

### Study Type

We used an exploratory qualitative design to examine stakeholder perceptions of digital tools, cross-sector collaboration, and data governance [30].

### Scope of the Study

The study took place in Kinshasa, the capital of the DRC, which serves as the country’s primary digital hub. The city accounts for approximately 40% of the national telecommunications market and has an estimated mobile penetration rate of 126% and a mobile internet access rate of 63.9% [26]. However, this apparent hyper-connectivity hides a more fragile reality, marked by network instability, reliance on multiple network operators to ensure adequate coverage for effective communication, and persistent energy limitations.

**Figure 1:**
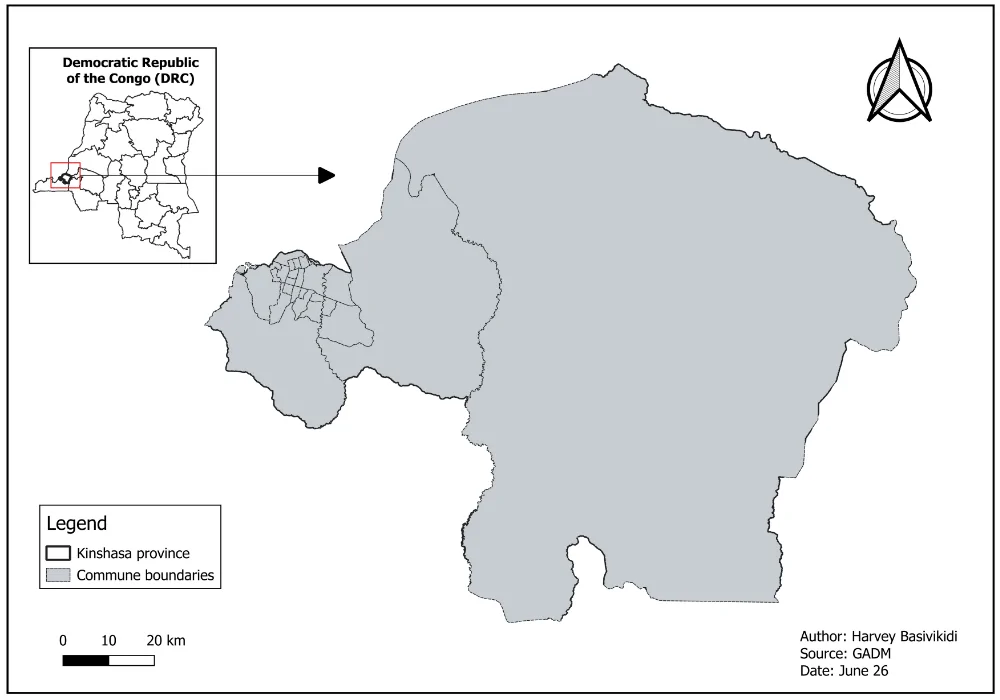
Geographic location of the study setting in Kinshasa, Democratic Republic of Congo. The map shows Kinshasa Province and its commune boundaries, with the inset map indicating its location within the Democratic Republic of Congo.

Figure 1 provides a general overview of the study setting in Kinshasa.

At the institutional level, the DH ecosystem is characterized by a coexistence of sector initiatives that lack interoperability, limiting the seamless circulation of data and the development of an integrated vision of health [21,22].

### Study Population and Sampling

#### Target Population

The target population consisted of professionals in Kinshasa working in the human, animal, environmental, and DH sectors who were involved in the use, management, production, or circulation of digital data and tools. Inclusion and exclusion criteria are given in Textbox 1.

**Inclusion and exclusion criteria.**

Participants included in the study were those who:

- Worked in one of the health sectors (human, animal, and environmental) or in the digital field.
- Had varying levels of experience in the use, management, or production of data or digital tools.
- They were primarily involved in operational, coordination, or mid-level management and planning functions within their respective areas.
- Provided free and informed consent for their participation in the study.

Excluded from the study were:

- Individuals who were no longer engaged in professional activity at the time of the study.
- Persons who refused to participate or consent to the interview.

#### Sampling

Purposive sampling was used for the relevance of the information [31]. The diversification of the sample was based on three dimensions:

1. The sector of activity (human, animal, environmental, or DH). The participant from the DH sector was included primarily to provide technical insights on digital infrastructure and interoperability. The other sectors were prioritized as they represent the main population responsible for implementing the ODH framework in the DRC.
2. The institutional level (central, operational, and community).
3. The type of role (coordination, management, and operational).

The sampling strategy intentionally prioritized stakeholders involved in operational and mid-level coordination roles. This choice was motivated by the study’s objective to capture real-world practices, constraints, and day-to-day interactions with digital tools across sectors. Operational actors are directly exposed to interoperability challenges, infrastructural limitations, and practical implementation barriers, making them key informants for assessing the feasibility of ODH in context. While some participants held coordination or management roles, the study did not specifically aim to represent high-level strategic decision-makers.

The deliberate exclusion of high-level strategic actors from the inclusion criteria reflects the study’s focus on operational implementation dynamics rather than policy design. This choice was motivated by the need to document a systematically underrepresented perspective in the ODH literature, while recognizing that governance-level voices are essential for a complete understanding of ODH feasibility and will be prioritized in future research.

### Data Collection

Semistructured interviews were conducted between 10 and 25 November 2025. They were guided by five thematic axes aligned with the study objectives: participants’ institutional role within the OH framework, perceptions of digital maturity and strategic vision of digital tools, current operational digital practices, perceived technical, human, and institutional barriers to interoperability, and perspectives on the feasibility of implementing ODH in the Congolese context.

Interviews lasted up to 45 minutes and were conducted in person or via Zoom [32] depending on participant availability [33].

Interviews were conducted in French, with clarifications in Lingala (a DRC *lingua franca*) when needed. Coding and interpretation were also conducted in French, and selected excerpts were translated into English for reporting. Key medical informatics and DH terminology were harmonized using the Medical Informatics and Digital Health Multilingual Ontology (MIMO) [34] to ensure consistency across interviews. MIMO was applied to map participant expressions to standardized DH and OH terms, improving the accuracy of thematic coding and enabling cross-sector comparability. Audio recordings were made and accurately transcribed. If recording was declined, detailed notes were taken with prior consent. All data were securely stored on a protected hard drive to ensure confidentiality and integrity.

The interview guide was adapted according to participants’ institutional level (central, operational, and community) to ensure contextual relevance. Before data collection, the guides were pretested with a small group of professionals from similar backgrounds to refine clarity and flow. All interviews were conducted by only one interviewer, ensuring consistency in data collection. Regular debriefing and iterative adjustments of probing techniques were used to maintain coherence across interviews and reduce interviewer variability.

### Targeted Document Review

A nonsystematic literature review complemented the interviews, contextualizing the policy and institutional frameworks. It focused on:

1. The National Plan for the Development of Health Information Systems (called PNDIS II) [18],
2. The National Digital Plan [35],
3. The National Strategic Development Plan 2024-2028 [20],
4. The One Health Strategic Plan 2022-2027 [36],
5. The IDSR 3 [23] and IADSR [24] guides.

The selected documents met all the following criteria:

1. Published by Congolese public institutions,
2. Published between 2018 and 2025,
3. Available in French and with official access.

### Data Analysis

The data were analyzed by an inductive thematic analysis inspired by Braun and Clarke [37] and Miles and Huberman [38]. Braun and Clarke’s framework guided the identification and interpretation of recurring topics across interviews through a six-step process, from familiarization to topic definition. Miles and Huberman’s approach provided practical tools for coding, data reduction, and cross-case comparison, including matrices to visualize patterns and relationships. By combining these frameworks, we ensured both interpretative depth and structured organization of qualitative data. The transcripts were imported into NVivo 15 (Lumivero) for encoding [39].

The transcripts were analyzed in their original language (French), which was also the primary language of the interviews. English was used only at the manuscript writing stage, where selected excerpts were translated for reporting purposes.

Saturation was assessed iteratively throughout data collection. After each interview, the coding structure was reviewed for emergent themes. Saturation was considered achieved when no new codes emerged over three successive interviews and when existing thematic categories were sufficiently dense and consistent across sectors and institutional levels.

The analysis was processed following six steps:

1. An initial phase of in-depth familiarization with the interview transcripts.
2. Open coding to identify 46 initial codes.
3. Development of a preliminary codebook: classification of the 46 keywords (of step 2) into intermediate categories.
4. Axial coding: Initial categories from open coding were grouped and linked to form broader topics, enabling the identification of patterns and relationships among concepts for a structured understanding of the data.
5. Structuring the codes into seven families: perceptions, digital practices, obstacles, data governance and FAIR, OH collaboration, opportunities, and recommendations.
6. Synthesis of the findings into three major topics directly related to the research objectives.

The “Technology Acceptance Model” (TAM) [40] and the “Unified Theory of Acceptance and Use of Technology” (UTAUT) [28] frameworks were used to guide coding and interpretation of participants’ attitudes toward digital tools. TAM helped contextualize perceived usefulness and ease of use, while UTAUT constructs such as performance expectancy and social influence explained patterns like anxiety or reluctance. These frameworks informed coding without constraining the inductive approach, capturing both expected and emergent factors affecting technology adoption.

### Quality Assurance and Validation

To ensure the integrity and rigor of the analytical process, we used several validation measures. Methodological triangulation was achieved by cross-referencing interview data with strategic documents, providing a multifaceted perspective on the research phenomenon. The analysis used a systematic, iterative coding approach across 3 distinct cycles to ensure thematic consistency.

To maintain transparency and accountability, a comprehensive audit trail was preserved through Excel matrices and NVivo exports. Furthermore, providing a thick description of the research context and participant profiles enhances the credibility, dependability, and transferability of the findings [41]. Finally, the study adheres to the COREQ (Consolidated Criteria for Reporting Qualitative Research) guidelines to ensure standardized and transparent reporting (Multimedia Appendix 1) [42].

Reflexivity was considered throughout the research process. The research team brings together complementary disciplinary backgrounds spanning global health, biomedical and health informatics, DH, epidemiology, OH, and ODH [13]. Team members have a local and international understanding of these research challenges. This interdisciplinary, international, and cross-institutional composition was treated as an analytical asset, enabling multiple interpretive lenses to be brought to bear on the data. At the same time, the team remained attentive to how prior expertise, particularly in OH frameworks, DH governance, and FAIR data principles, may have shaped the framing of research questions, the interpretation of findings, and the proposed conceptual model. To mitigate such risks, analytical decisions were documented systematically, coding processes were discussed iteratively across the team, and a deliberate effort was made to foreground participant voices in the presentation of results rather than prematurely mapping findings onto existing theoretical constructs.

### Ethical Considerations

The protocol has been approved by the National Committee on Health Ethics (N°725/CNES/BN/PMMF/2025). Participation was voluntary. Oral or written informed consent was obtained before each interview. Anonymity and confidentiality are guaranteed through alphanumeric codes and secure data storage.

## Results

### Overview

Inductive thematic analysis identified three main topics:

1. Perceptions and representations of DH technology.
2. Conditions, constraints, and structural factors influencing digitalization and interoperability.
3. The necessary conditions for the operational implementation of an ODH approach integrating the FAIR principles.

### Characteristics of the Participants

The study included 22 participants from the human, animal, environmental, and DH sectors in Kinshasa, DRC. Participants were recruited purposively; initial contacts were identified among key professionals, who then facilitated introductions to additional relevant stakeholders. Interviews were scheduled based on participants’ availability and conducted directly by a qualified researcher (HB), enabling engagement across different roles while maintaining independence.

The majority of participants came from human health (n=9, 40.9%) and animal health (n=8, 36.4%), with fewer from environmental health (n=4, 18.2%) and a single DH expert (n=1, 4.50%). The inclusion of one digital expert was intended to provide specialized technical insights into digital systems and interoperability. In contrast, the remaining participants represented the primary users of DH tools. Participants were predominantly male (n=15, 68.2%) and mostly aged 35 to 49 (n=9, 40.9%), followed by those aged 34 and under (n=8, 36.4%) and those aged 50 and older (n=5, 22.7%).

From a role-type perspective, half of the participants were in operational positions (n=12, 54.5%), followed by 31.8% (n=7) at the central level and 13.7% (n=3) at the community level. All participants provided informed consent before the interviews (Table 1). The imbalance in sector representation, particularly the inclusion of only an ODH expert, is acknowledged as a limitation. This participant was not intended to represent the broader community’s adoption of ODH, but rather to provide specialized technical insights into digital systems and interoperability challenges.

**Table 1:** Socio-professional characteristics of the interviewees.

Variables			Participants (N=22), n (%)
Sex			
	Male	15 (68.2)	
	Female	7 (31.8)	
Age (years)			
	35-49	9 (40.9)	
	≤34	8 (36.4)	
	≥50	5 (22.7)	
Industry			
	Human health	9 (40.9)	
	Animal health	8 (36.4)	
	Environmental health	4 (18.2)	
	Digital	1 (4.50)	
Hierarchical level and type of role			
	Central and decision-making actor	7 (31.8)	
	Operational and technical professional	12 (54.5)	
	Community and field actor	3 (13.7)	

To better assess the functional and institutional diversity of the sample, a matrix was developed that cross-referenced the activity sector, the institutional level, and the hierarchical level and role type (Table 2). This matrix shows the intentional structuring of the population studied across three complementary dimensions. It highlights the joint presence of decision-making actors, technical professionals, and field actors, enabling documentation of digitalization and the OH approach at different levels of the system.

Thus, this study, therefore, focused on understanding perceptions, practices, and adoption among nondigital professionals, while the digital expert served to contextualize the technological environment.

The average interview duration was 29 minutes (SD 11 minutes and 20 seconds).

**Table 2:** Diversification matrix of participant profiles.

Hierarchical level and type of role	Animal health (n=8, 36.4%)	Environmental health (n=4, 18.2%)	Human health (n=9, 40.9%)	Digital (n=1, 4.50%)
Central and decision-making actor (n=7, 31.8%)	Coordinator (n=1, 12.5%)	Director, program expert (n=2, 50%)	Coordination and management of health data (n=4, 44.5%)	—^a^
Operational and technical professional (n=12, 54.6%)	Management and operational implementation (n=6, 75%)	Professor (n=1, 25%)	Physician, laboratory technician (n=4, 44.5%)	Software engineer (n=1, 100%)
Community and field actor (n=3, 13.6%)	Farmer (n=1, 12.5%)	Farmer (n=1, 25%)	Community relay (n=1, 11%)	—

^a^Not applicable.

### Topic 1: Perceptions and Representations of DH

This topic explores how actors envision, understand, and engage with DH in their daily professional practices. The results highlight both positive representations associated with modernizing work. However, there are mixed feelings about the challenges of understanding and appropriating this concept.

### Individual Perceptions, Understanding, and Affective Relationship to Digital Technology

For most participants, digital technology is not perceived as a simple technical tool, but as a marker of modernity and a factor in the transformation of work. Stakeholders often associate digitalization with increased efficiency, better information organization, and the alleviation of constraints associated with manual file management.

A physician in the human health sector describes the contribution of digital technology in patient management as follows:

All of our patients’ files are encoded in the machine... it allows us to go a little faster than if we had to look, especially patients who have chronic problems; the sick who come back often.

P04, Human Health, Physician

Beyond functional use, some participants express a strong emotional attachment to digital technology, associating it with their professional and generational identities. Digital technology then becomes a symbol of belonging to technological modernity.

I’m a total fan of digital technology. I’m from the digital generation. I am from the generation of artificial intelligence. I can’t be indifferent to digital technology.

P09, Animal Health, Veterinarian

However, this positive relationship is not homogeneous. Several participants highlighted difficulties with appropriation, particularly among older agents, for whom using digital tools can be a source of anxiety or exclusion.

...We still have staff who are sometimes quite old and who are not used to smartphones and tablets.

P10, Human Health, Division Manager

These results show heterogeneity in relationships with digital technology, ranging from enthusiastic adherence to a form of distance or reluctance linked to age and technological experience.

### Digital Practices, Fragmentation, and Local Appropriations

**Figure 2:**
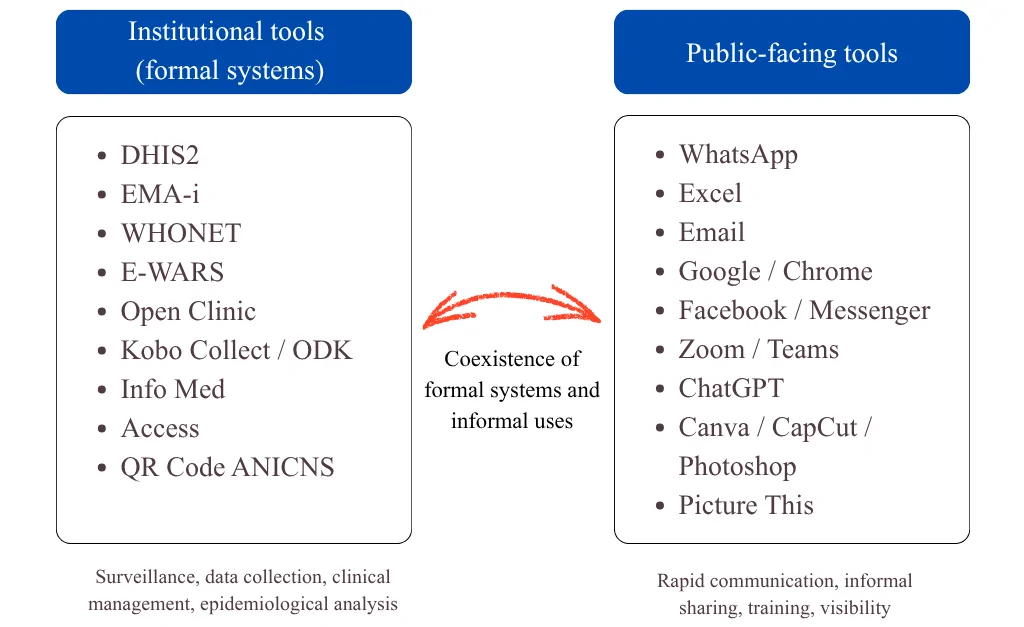
Typology of digital tools used by One Health actors in Kinshasa, Democratic Republic of Congo. DRC: Democratic Republic of Congo.

The interviews highlight the coexistence of formal institutional digital systems and informal practices of appropriating digital tools (Figure 2). Participants described using official platforms such as DHIS2, electronic medical records, and specialized databases, while highlighting the persistence of paper in daily practice.

In many contexts, data is still collected manually before being entered into digital systems, which is perceived as an additional workload.

...It’s really a notebook. There are notebooks from 1900... When a piece of data arrives, we write the date, record it, and it passes.

P13, Animal Health, Veterinarian

This double entry is often described as tedious.

...It’s really a tedious exercise... Asking them to go back to the tools, correct, and recorrect in DHIS2 again isn’t easy.

P14, Human Health, Program Officer

Digital practices are thus divided between mandatory institutional use for reporting and significant use of digital tools by the general public, particularly for communication and the exchange of information between peers.

So for us to use the data, we enter DHIS2 and then we extract, we do the analyses...

P14, Human Health, Program Officer

...Digital tools help us to get in touch with other colleagues. […] when we exchange on WhatsApp, they also give us their experiences...

P11, Animal Health, Veterinarian

Overall, the participants described a pragmatic appropriation of digital technology: tools are used where they are perceived as useful, fast, and accessible, while institutional platforms are sometimes experienced as cumbersome, complex, or poorly adapted to realities on the ground.

These results show that, despite an overall positive perception of digital technology, its integration into professional practices remains marked by fragmented systems, paper-digital coexistence, and a strong dependence on informal communication tools.

### Topic 2: Structural and Institutional Impediments to Interoperability

This topic brings together the material, human, and organizational elements that shape the effective use of digital technology and the potential for interoperability between sectors. Participants describe persistent structural constraints limiting the functionality of digital tools and their integration into OH logic.

**Figure 3:**
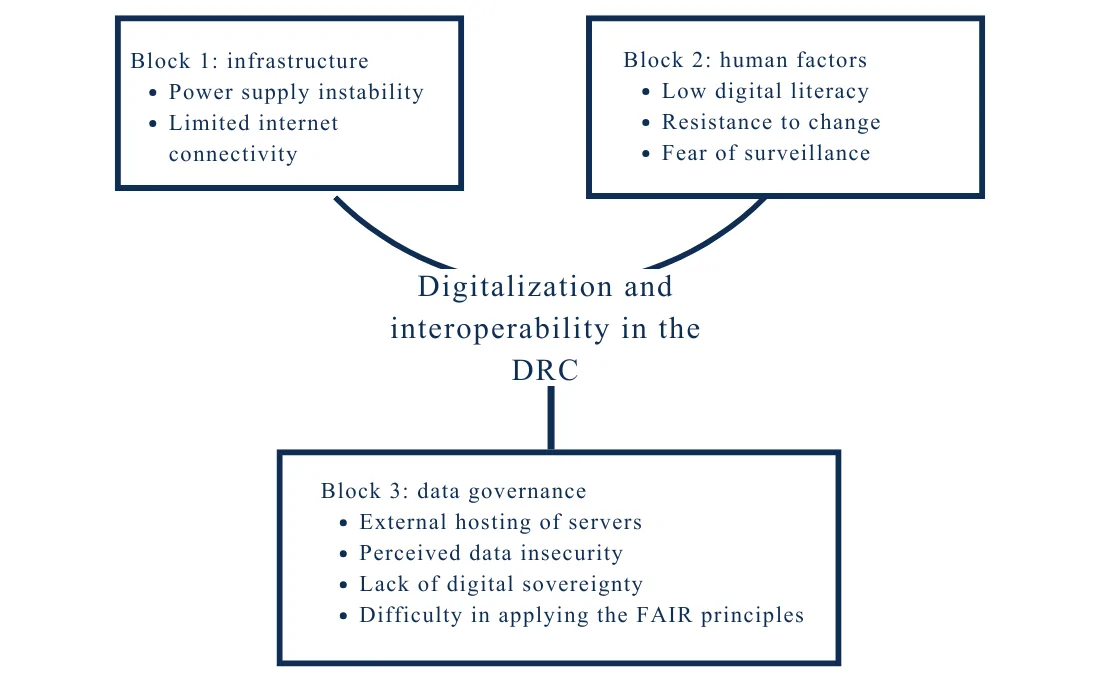
Architecture of the structural constraints of digitalization and interoperability in the DRC. DRC: Democratic Republic of Congo; FAIR: findable, accessible, interoperable, reusable.

Figure 3 summarizes these constraints by showing that the challenges of DH in the DRC stem from the interaction among three main blocks: hardware infrastructure, human factors, and data governance.

### Technical and Infrastructural Barriers

Electricity instability and poor internet coverage are major obstacles to digitalization, especially in rural areas. Several participants indicated that the absence of these prerequisites makes digital tools unusable in many contexts.

There are places where there is no electricity, and there is no connection, and all that…

P11, Animal Health, Veterinarian

But here, we don’t even have electricity. How do you want us to be in the digital world?

P16, Animal Health, Breeder

These speeches show that the use of digital technology depends directly on basic material conditions that are not guaranteed uniformly across the territory.

### Sociotechnical and Human Barriers

Participants report a lack of technical training and limited mastery of digital tools among some staff, which limits the adoption of these tools.

The obstacles can be first and foremost on the service provider’s side, the use of phones and tablets, because not everyone is... well-versed in the use of digital equipment…

P10, Human Health

Some participants also report mistrust of digitalization, linked to fears of surveillance or data security.

...And a few more services... who refuse to digitize their file. They want to stay with the hard file because they don’t trust our security system…

P04, Human Health, Physician

These elements show that the barriers to digitalization are not only technical, but also human and organizational.

### Institutional and Governance Constraints

Several participants pointed out that external hosting of servers raises concerns about national sovereignty and data security.

...These are solutions that are foreign. These are mostly American solutions. The retrieval of information in these platforms is subject to American laws

P18, Digital, IT

The question of data security and durability is also recurrent in the speeches.

...It’s not safe for data storage... Is there a bug? Is there a hack? Will our data survive?

P04, Human Health, Physician

Overall, participants agreed that electrical instability, limited connectivity, and reliance on external solutions are major structural constraints on digitalization and the effective application of FAIR principles.

### Topic 3: Conditions Necessary for the Operational Implementation of ODH, Incorporating the FAIR Principles

This topic brings together the elements that participants consider essential for making an ODH approach functional in the Congolese context.

### OH Collaboration and Relationship Challenges

Cross-sectoral collaboration is often constrained by power relations and symbolic hierarchies between sectors, particularly to the detriment of animal and environmental health.

...The human side takes over and takes the lion’s share... they leave the least to the vets…

P19, Animal Health, Accounting

...There are human doctors who think they are superior to veterinarians…

P11, Animal Health, Veterinarian

### Opportunities to Improve Public Health

Digital technology is seen as a way to strengthen cross-sector collaboration and better anticipate health risks.

Digitalization can facilitate collaboration [...] Animal health plays the role of sentinel in relation to human health…

P20, Human Health, Coordinator

### Human Capabilities and Digital Literacy

Participants stressed the need to strengthen human skills to enable sustainable ownership of ODH.

The One Health concept still has a very long way to go: it is to train the epidemics, to train the different doctors, to train the different experts…

P20, Human Health, Coordinator

The training is described as going beyond the one-off seminar framework.

### Interoperability and Infrastructure: The Material Foundation of the FAIR Approach

Interoperability depends on stable infrastructure and solutions adapted to the local context.

The main problem... is this concern for the lack of digital solutions compatible with the realities that we are experiencing here in the DRC.

P18, digital, IT

We should develop bridges between the tools…

P01, Animal Health, Head of Department

### Strategic Recommendations and Institutional Levers

The need for strong leadership is widely shared.

...We have to create a framework... at the level of the Presidency or the Prime Minister…

P20, Human Health, Coordinator

### Narrative Synthesis According to the Three Analytical Axes

**Figure 4:**
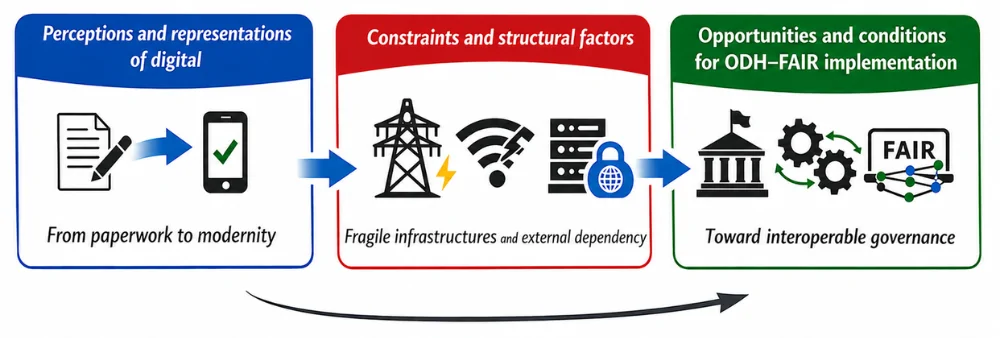
Conceptual model of One Digital Health operationalization in the Democratic Republic of Congo, integrating (findable, accessible, interoperable, reusable) FAIR principles. The model illustrates the interplay between stakeholder perceptions and digital practices, structural and infrastructural constraints, and the essential conditions for One Digital Health implementation. Three pillars identified by participants: energy and connectivity, human capacity building, and system interoperability form a “digital bridge” connecting the human, animal, and environmental health sectors, facilitating coordinated surveillance and collaboration. FAIR: findable, accessible, interoperable, reusable. ODH: One Digital Health.

Figure 4 illustrates the articulation among digital perceptions and practices, structural constraints, and the conditions necessary for implementing ODH.

It represents the implementation of ODH in the DRC as a digital bridge connecting the human, animal, and environmental sectors, based on three pillars identified by the participants: energy and connectivity, human capacity building, and system interoperability.

In this context, the DH campus project is mentioned in the PNDIS II.[18] appears to be a relevant structural response to the institutionalization of intersectoral and continuing training. Without sustainable investment in training and collaborative governance, the ODH risks remaining an ambitious conceptual framework that is difficult to apply in the Congolese context.

## Discussion

### Main Findings

Our study identifies three critical dimensions affecting the implementation of ODH in the DRC.

The first dimension is a dual-track digital reality. While digital tools such as DHIS2 and sectoral platforms are acknowledged as catalysts for modernization, their formal use is often overshadowed by what is termed “informal adaptability.” Practitioners frequently circumvent institutional gaps by using private message platforms (eg, WhatsApp), creating tension between immediate operational efficiency and long-term concerns about data security and confidentiality.

The second identified dimension concerns “infrastructures and data sovereignty bottlenecks.” Indeed, digitalization is currently an “add-on” rather than a replacement for paper, leading to a double workload and “e-chaos.” Success is fundamentally bottlenecked by unstable energy and connectivity, as well as significant concerns about digital sovereignty and the external hosting of national health data.

Finally, the third and most critical dimension concerns “siloed intersectionality.” Although digital technology is perceived as a potential bridge between sectors, current efforts are hampered by “digital islands.” Institutional hierarchies and power dynamics between disciplines prevent the FAIR principles from being operationalized.

### Sociotechnical Synthesis and Theoretical Implications

#### Behavioral Appropriation and the Cognitive Divide

Digital technology is mainly perceived as an instrument of modernity and efficiency, improving access to information and reducing administrative burdens. This perceived utility confirms technology acceptance patterns, which identify utility as a central determinant of adoption [28].

The speeches also reveal a symbolic dimension of digital technology, associated with professional and generational identity. This reading aligns with the idea that, in Africa, digital technology is as much a “state of mind” as a technical tool [43]. However, this appropriation remains unevenly distributed. Older agents express more anxiety and reluctance, which corresponds to UTAUT models highlighting the role of perceived skills and trust in the tool [28].

Thus, the observed digital divide appears less a matter of material access than of a cognitive and professional divide, linked to skills, training, and perceived system security. This interpretation is consistent with the notion of “eHealth literacy” developed by Norman and Skinner [27].

Finally, the widespread use of consumer tools such as private messaging platforms like WhatsApp illustrates participants’ strong ability to adapt to the limitations of institutional platforms. However, these informal practices raise significant ethical concerns regarding confidentiality and illustrate institutional fragility, in which the continuity of systems depends more on individual initiative than on solid organizational structures [12]. For the ODH, the acceptance of digital technology must therefore be accompanied by secure institutional frameworks and structured human capacity building.

#### Infrastructural Realities and the Sovereignty Challenge

The results confirm that energy and connectivity are the main bottlenecks to digitalization in the DRC. The absence or instability of electricity and the Internet makes digital tools ineffective, especially outside urban centers. This situation illustrates the gap between ambitious digital policies and material realities on the ground, described as the “rural myth versus urban reality” [10].

The national data also highlight that low energy coverage is a major structural constraint for any DH strategy [20]. The digital transformation thus appears inseparable from a robust energy policy, calling into question a strictly technological vision of digitalization.

The coexistence of paper and digital generates a double workload and confirms that digitalization tends to add to existing practices rather than replace them [12,44]. This overlap promotes the fragmentation of initiatives and feeds the risk of “e-chaos,” characterized by the proliferation of noninteroperable tools [11].

In addition, concerns about external data hosting underscore the strategic issue of digital sovereignty. This dependency aligns with UNESCO’s analyses of the fragility of data ecosystems in LMICs [45]. It is also consistent with the guidelines of the Plan national du numérique (PNN) and PNDIS II, which recommend establishing sovereign national data centers [18,35].

From the perspective of ODH and the FAIR principles, interoperability cannot be effective without a stable, secure, and sovereign national infrastructure that guarantees the accessibility, reusability, and governance of data [16].

#### Paths Toward Integrated Governance and ODH

The results show that relational and institutional barriers across sectors initially hamper the ODH. Symbolic tensions and the implicit hierarchization of disciplines confirm that intersectoral collaboration cannot be taken for granted. This observation is consistent with the analyses of Zinsstag et al [46], which identify power relations and institutional compartmentalization as major obstacles to the OH approach. It is also in line with the findings of the DRC’s OH strategic plan 2022-2027, which highlights weaknesses in operational coordination mechanisms [36].

At the same time, participants perceive digital technology as a lever to overcome this compartmentalization by facilitating the circulation of information and the development of a shared vision of health risks. This reading is consistent with ODH’s conceptual framework, which defines digitalization as a catalyst for OH integration [13]. However, this promise remains conditional on the effectiveness of interoperability. Without technical bridges between sectoral systems, data remains fragmented into “digital islands,” rendering the FAIR principles, in particular interoperability and reusability, inoperative [16].

Finally, the emphasis on training confirms that the success of the ODH is largely based on the development of human capacities. Digital transformation cannot be reduced to a question of equipment; it requires an evolution in skills and professional cultures. This conclusion aligns with the recommendations of Bhutta et al [47] on health education reform and with Norman and Skinner’s expanded definition of digital literacy [27].

### Strengths and Limitations

This study is a pioneering multisectoral analysis in DRC, particularly for its empirical application of the ODH framework from a FAIR principles’ perspective. This research moves beyond purely theoretical models to document real-world implementation challenges in an LMIC.

A primary strength lies in the methodological triangulation used. By combining a diversified qualitative sample with perspectives from human, animal, and environmental health sectors, the study captures the “symbolic dimension” of digital tools that quantitative surveys often miss. This approach enabled the identification of the “cognitive divide” and “institutional fragility,” providing a nuanced understanding of how professional identity and generational shifts affect technology adoption. Furthermore, the inclusion of both formal systems (DHIS2) and informal communication practices (eg, WhatsApp) provides a highly realistic map of the digital ecosystem, offering a unique contribution to the PNN and PNDIS II evaluations.

Despite these contributions, several limitations must be acknowledged. First, the geographic scope was primarily concentrated in Kinshasa. While Kinshasa serves as the institutional hub, the findings may not fully capture the extreme infrastructural constraints found in deep rural provinces, where the “rural myth” of digitalization faces even harsher material realities. Consequently, the results offer a “centralized” view of the DRC’s DH landscape that may not fully generalize to the entire national territory.

The study primarily included operational and mid-level stakeholders, which provided valuable insights into day-to-day implementation challenges but limited the analysis of higher-level governance, policy coordination, and strategic decision-making. Therefore, the operational focus was a deliberate design choice, rooted in the study’s primary aim to capture ground-level, real-world experiences of digital tool integration across sectors that are often invisible in policy discourse yet determinative of implementation outcomes. As Braun and Clarke’s framework and our purposive sampling rationale make clear, operational actors represent the primary interface between institutional aspirations and daily practice. In a context such as Kinshasa, where the distance between policy design and field implementation is well documented, this perspective carries significant empirical weight. Nevertheless, the strategic and governance frameworks were not absent from the study; they were addressed through a targeted document review that examined the PNDIS II, the PNN, the One Health Strategic Plan 2022-2027, and related regulatory texts. This layer of analysis provided the institutional and policy backdrop against which operational perceptions were interpreted. Future studies should adopt a multilevel sampling strategy that integrates high-level governance and strategic decision-makers alongside operational actors to assess the coherence or disconnect between institutional ambitions and ground-level realities in the operationalization of ODH.

While the sample was diverse, only a single DH expert was included. This participant provided technical insights on system architecture and interoperability, rather than representing broader adoption of the ODH framework, which remains primarily informed by human, animal, and environmental health professionals. The absence of direct field observations and in-depth technical system audits means that the specific performance of software platforms and the exact nature of data silos cannot be technically measured. Finally, the study focuses on a specific point in time; given the rapid evolution of DH policies in the DRC, the findings reflect a snapshot of a transition period that may shift as new sovereign data centers are established.

### Conclusions

This study reveals a crucial insight: the success of the ODH approach in LMICs hinges not just on access to digital tools, but fundamentally on the robustness of their structural, institutional, and human foundations. In Kinshasa, while digital technology is viewed as a powerful catalyst for modernizing and enhancing professional practices, its true potential is severely constrained by energy instability, fragmented information systems, weak intersectoral governance, and stark disparities in digital skills. These challenges must be addressed to unlock the transformative power of technology. Thus, future DH strategies in the DRC should move toward implementing standardized reference architectures. Specifically, integrating the DH domains through a cloud-edge approach may support improved data interoperability while addressing the systemic connectivity constraints identified in this study [48].

## Appendix

Multimedia Appendix 1 COREQ checklist.

## Abbreviations

COREQ: Consolidated Criteria for Reporting Qualitative Research
DH: digital health
DHIS2: District Health Information Software 2
DRC: Democratic Republic of the Congo
FAIR: findable, accessible, interoperable, reusable
GDP: gross domestic product
IADSR: Integrated Animal Disease Surveillance and Response
ICCN: Institut Congolais pour la Conservation de la Nature (in English; “Congolese Institute for Nature Conservation”)
IDSR: Integrated Disease Surveillance and Response
LMIC: low- and middle-income country
MIMO: Medical Informatics and Digital Health Multilingual Ontology
ODH: One Digital Health
OH: One Health
PNDIS: Plan national de développement de l’informatique de santé (in English; “National plan for the development of health informatics”)
PNN: Plan national du numérique (in English; “National Digital Plan”)
TAM: Technology Acceptance Model
UG PDSS: Unité de Gestion du Projet de Développement du Système de Santé (in English; “Health System Development Project Management Unit”)
UNESCO: United Nations Educational, Scientific, and Cultural Organization
UTAUT: Unified Theory of Acceptance and Use of Technology

## Data Availability

The qualitative data generated and analyzed in this study are not made public for ethical reasons related to participant confidentiality. Anonymized extracts may be provided by the corresponding author upon reasonable request and subject to appropriate ethical authorization.
