# Supplementary material for: Operationalizing One Digital Health and FAIR Data Principles for Disease Surveillance in a Low-Medium Income Country in Africa: Qualitative Study in the Democratic Republic of the Congo
Source: JMIR Med Inform. 2026 Aug 5;14:e93321. doi: 10.2196/93321 (PMC13490936; doi:10.2196/93321)
Supplement: Supplementary file 1 [file medinform_v14i1e93321_app1.docx]

**COREQ – Verification**

Domain 1: Research team and reflexivity

1. Interviewer

Harvey Basivikidi conducted all interviews

2. Qualifications

Doctor of Medicine, Master's student in Disease Ecology, Natural Hazards and Risk Management

3. Profession

Doctor

4. Gender

Male

5. Experience and training

Training in research methods, health systems research, One Health, experience in field data collection in academic and professional projects.

6. Relationship established

A professional relationship was established prior to data collection with some participants in academic and institutional collaboration, but not with all participants.

7. Participants' knowledge of the interviewer

Participants were informed about the researcher's academic background, the objectives of the study and its relevance for strengthening digital health and One Health in the DRC

8. Investigator Characteristics

The interviewer is very interested in digital health and the One Health approach, which may have influenced the direction of the discussions. Reflexivity was maintained in order to minimize bias by using a semi-structured guide and triangulation with strategic documents.

Domain 2: Study Design

9. Methodological orientation and theory

Qualitative scoping study using thematic analysis, building on the One Digital Health framework and the principles of One Health and digital health governance.

10. Sampling

Interest-based sampling.

11. Approach

Participants were approached through professional networks, institutional contacts and direct face-to-face or electronic communications.

12. Sample size

A total of 22 participants were interviewed

13. Non-participation

Some invited participants declined the invitation or were not available due to work and time constraints.

14. Data Collection Framework

The interviews were all conducted in professional settings (offices, institutions) and, when necessary, online (Zoom).

15. Presence of non-participants

No non-participants were present during the interviews

16. Sample Description

Participants included professionals from the human, animal, environmental and digital health sectors, involved in the policymaking, implementation, research and management of health systems in Kinshasa.

Data collection

17. Care Guide

A semi-structured interview guide was developed based on the One Digital Health framework and tested prior to full data collection.

18. Repeat interviews

No repeated maintenance was conducted

19. Audio recording

Interviews were recorded with the consent of the participants

20. Field Notes

Field notes were taken during and after the interviews to capture contextual elements and analytical first impressions.

21. Duration

Interviews lasted an average of 35 minutes

22. Data saturation

Data collection continued until thematic saturation was reached.

23. Restitution of transcripts

Transcripts were not returned to participants for validation

Domain 3: Analysis and results

24. Number of Data Encoders

Primary coding was done by Harvey Basivikidi, with methodological guidance from the INOHA teaching team and supervisors.

25. Description of the coding tree

A formal coding tree has been developed, but is not presented graphically in the manuscript.

26. Derivation of Themes

The themes were derived inductively from the data using thematic analysis.

27. Software

Qualitative data were managed using NVivo 15 software.

28. Verification by participants

Participants did not provide direct feedback on the results

Report

29. Featured quotes

Participant quotes are presented in the "Results" section and identified by participant codes.

30. Consistency of data and results

The results are consistent with the data presented and supported by textual citations.

31. Clarity of the main themes

The main themes are clearly presented and structured according to the analytical framework.

32. Clarity of sub-themes

Secondary themes and divergent perspectives are addressed where relevant.
